# Transcriptomic-based analysis of endometrial tissues from adenomyosis patients reveals significant inflammation biomarkers: A bioinformatics study

**DOI:** 10.18502/ijrm.v23i5.19265

**Published:** 2025-07-29

**Authors:** Marni Sianturi, Alauddin Syaifulanwar, Darmawi Darmawi, Wirawan Adikusuma, Lalu Muhammad Irham, Muhammad Yusuf, Rifia Tiara Fani, Febriani Febriani

**Affiliations:** ^1^Master's Program in Biomedical Sciences, Faculty of Medicine, Universitas Riau, Pekanbaru, Indonesia.; ^2^Department of Pharmacology, Faculty of Medicine, Universitas Awal Bros, Pekanbaru, Indonesia.; ^3^Department of Histology, Faculty of Medicine, Universitas Riau, Pekanbaru, Indonesia.; ^4^Department of Pharmacy, Universitas Muhammadiyah Mataram, Mataram, Indonesia.; ^5^Research Center for Computing, Research Organization for Electronics and Informatics, National Research and Innovation Agency (BRIN), Cibinong Science Center, Cibinong, West Java, Indonesia.; ^6^Department of Pharmacology and Clinical Pharmacy, Universitas Ahmad Dahlan, Yogyakarta, Indonesia.; ^7^Department of Obstetrics and Gynecology, Faculty of Medicine, Universitas Riau, Pekanbaru, Indonesia.; ^8^Department of Veterinary, Faculty of Medicine, Universitas Riau, Pekanbaru, Indonesia.

**Keywords:** Adenomyosis, Bioinformatics, Biomarker, Inflammation.

## Abstract

**Background:**

Adenomyosis is a gynecological disorder characterized by the presence of endometrial tissue within the myometrium, with incidence rates ranging from 10–65% among women of reproductive age.

**Objective:**

This study utilized transcriptomic analysis to identify significant biomarkers associated with inflammation in endometrial tissue from patients with adenomyosis.

**Materials and Methods:**

In this bioinformatics study, we utilized publicly available transcriptomic datasets. The research involved the systematic analysis of RNA sequencing data obtained from the NCBI-GEO database. Using a high-throughput RNA sequencing database from GSE190580 and GSE157718, we compared gene expression profiles between endometrium tissues of adenomyosis patients and healthy controls. Subsequently, pathways implicated in adenomyosis were analyzed through the Kyoto Encyclopedia of Genes and Genomes and gene ontology.

**Results:**

Pathway analysis revealed the aberration of inflammation-related pathways, including tumor necrosis factor (TNF) and Ras-related protein 1 signaling. Furthermore, gene ontology analysis uncovered key biological processes, such as macrophage differentiation and extracellular matrix organization, which are central to the inflammatory response in adenomyosis. Candidate biomarkers, including transmembrane protein kinases, were identified as potential therapeutic targets. We found the top 5 genes that play a role in inflammation in adenomyosis, including TNF-α-induced protein 6, matrix metalloproteinase 7, TNF-α-induced protein 3, leukemia inhibitory factor, and serum and glucocorticoid-regulated kinase 1. Statistical significance was determined with adjusted p < 0.05.

**Conclusion:**

These findings enhance our understanding of the molecular mechanisms of adenomyosis and propose novel biomarkers for more effective diagnostic and therapeutic strategies.

## 1. Introduction

Adenomyosis is a common benign gynecological disease typically characterized by the invasion of endometrial tissue into the myometrium (1). Epidemiological data indicate that women of reproductive age are at high risk of developing adenomyosis, with reported incidence rates ranging from 10–65% (2). The exact incidence and prevalence of adenomyosis in Indonesia are not well-defined due to the need for histological confirmation following clinical diagnosis based on symptoms and imaging (3, 4). In recent years, delayed reproductive age in women has led to an increased occurrence of adenomyosis in nulliparous women (5). The exact causes of adenomyosis are not fully understood; one theory suggests that adenomyosis develops when the basal layer endometrium invades and extends into the myometrium. Despite advances in science and technology, no single theory can fully explain the pathophysiology of adenomyosis (6).

The main symptoms in patients with adenomyosis include prolonged menstruation, increased menstrual volume, infertility, and progressive dysmenorrhea, among others, ultimately impacting the quality of patients (7). Dysmenorrhea, excessive menstrual bleeding, and infertility are likely caused by inflammation, neurogenesis, angiogenesis, and contractile abnormalities in the endometrial and myometrial components (8). Management in patients with adenomyosis is based on symptoms, age, and reproductive needs. Patients with mild symptoms, a desire for fertility, or approaching menopause may be given hormone therapy. Patients with severe treatment-resistant symptoms may require surgery (7). Definitive management for adenomyosis currently involves hysterectomy (9).

Historically, adenomyosis diagnosis has relied on invasive procedures such as histological analysis post-hysterectomy or imaging methods like ultrasound and MRI. These methods, however, have limitations in terms of sensitivity and specificity, particularly in the disease's early stages (10). Therefore, discovering accurate, non-invasive biomarkers is essential for enhancing early diagnosis and improving patient outcomes. Recently, bioinformatics has emerged as a crucial resource for identifying new biomarkers by leveraging large-scale genomic, transcriptomic, and proteomic datasets (11).

Bioinformatics combines computational methods with biological data to analyze intricate molecular networks and gene expression profiles. This technique enables researchers to systematically identify differentially expressed genes (DEGs), regulatory pathways, and molecular signatures linked to diseases like adenomyosis (12).

Several studies have successfully utilized bioinformatics to delve into the molecular mechanisms of adenomyosis. For instance, Wang et al. highlighted potential non-hormonal therapy targets by evaluating gene expression in both eutopic and ectopic endometrial tissues from patients with adenomyosis (13). Another study by Liu et al. analyzed microarray data to pinpoint dysregulated pathways involved in inflammation and angiogenesis, 2 critical processes in adenomyosis pathophysiology (14).

Employing high-throughput RNA sequencing and gene expression data from platforms like the Gene Expression Omnibus (GEO), we will explore the differential gene expression between adenomyosis and normal endometrial tissues. Through gene expression profiling and bioinformatics approaches, the study seeks to uncover molecular signatures and pathways that could shed light on the role of inflammation in the pathogenesis of adenomyosis, providing insights for potential therapeutic targets and diagnostic tools.

## 2. Materials and Methods 

This bioinformatics study utilized publicly available transcriptomic datasets. The research involved the systematic analysis of RNA sequencing data obtained from the NCBI-GEO database to investigate gene expression patterns and identify potential biomarkers and therapeutic pathways associated with adenomyosis. Our inquiry focused on “adenomyosis", aimed to uncover novel biomarkers for this condition and explore crucial therapeutic pathways associated with it. We initially explored the “human organism" dataset, starting with the keyword adenomyosis. Subsequently, RNA sequencing investigations were conducted, followed by gene expression analyses. Through data analysis, we identified 239 datasets mentioning “adenomyosis".

We obtained 19 datasets depicting gene expression patterns in adenomyosis. The inclusion criteria specified that RNA and high-throughput sequencing can be analyzed with GEO2R, focusing on adenomyosis and non-adenomyosis control samples.

**Figure 1:**
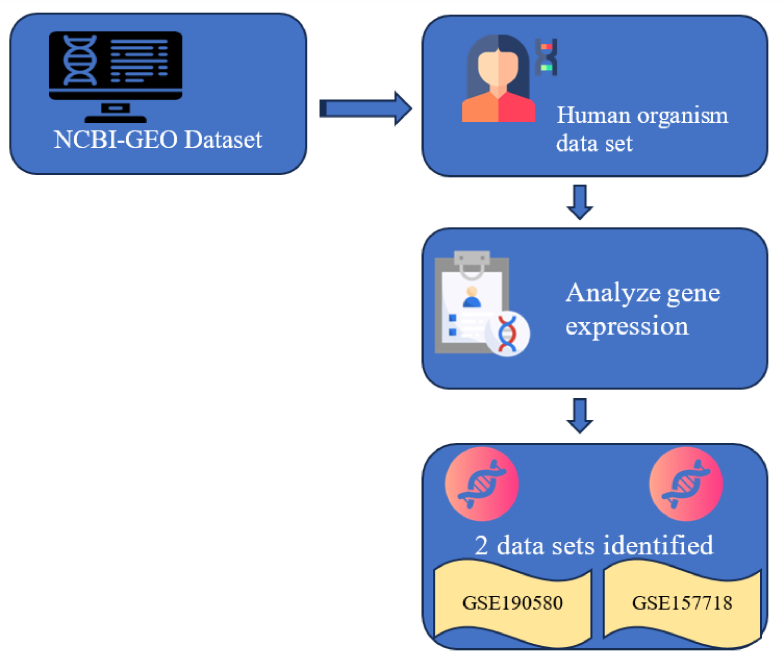
Flow diagram of the studies.

Exclusion criteria encompassed organoid, cell line, endometriosis, ovarian cancer, and leiomyoma. We excluded 17 datasets, resulting in the retention of 2 relevant datasets (Figure 1).

Ultimately, 2 datasets, GSE190580 and GSE157718, were selected for final analysis, as they met the inclusion criteria of high-quality RNA sequencing data focused on adenomyosis. According to GEO, GSE190580 pertains to RNA sequencing of eutopic endometrium and myometrium from women with and without diffuse adenomyosis in the proliferative phase of the menstrual cycle. At the same time, GSE157718 involves transcriptome analysis of eutopic endometrium stromal cells via RNA-sequencing in adenomyosis patients. Statistical significance was determined with an adjusted p < 0.05.

### Data and pathway analysis

A Venn diagram was generated to assess the overlap between the 2 datasets. From this analysis, 199 genes were identified (accessible at https://bioinformatics.psb.ugent.be), subsequently subjected to pathway analysis using the Kyoto Encyclopedia of Genes and Genomes (KEGG) via ShinyGO 0.77 (accessible at http://bioinformatics.sdstate.edu/go/). Through KEGG analysis, we delved into the biological pathways related to these genes, linking their expression profiles to understand their roles in adenomyosis. The KEGG pathway analysis highlighted 2 key signalling pathways: tumor necrosis factor (TNF) and Ras-related protein 1 (Rap1). Within the ShinyGO 0.77 platform, we specified the “human" species for the analysis of adenomyosis. This exploration visually illustrates the molecular mechanisms of inflammation in the pathogenesis of adenomyosis. Statistical assessments, including false discovery rate (FDR) adjustments performed using the Benjamini-Hochberg method, were conducted to refine p-values and minimize erroneous conclusions. A lower FDR signifies increased reliability in the results.

## 3. Results

The study selection flow diagram of literature retrieval is shown in figure 1. A total of 239 dataset studies were retrieved, and 220 were excluded through preliminary screening. The remaining 19 datasets were screened through species. In the process of full screening, 7 datasets were excluded because they were not derived from Homo sapiens samples. 4 were excluded because they could not be analyzed with Geo2R.

6 were excluded because of deep infiltrating adenomyosis, treatment medicine, organoid, no control group, ovarian cancer, and cell line. Finally, 2 datasets were included in this study.

The GEO database was used to collect transcriptomic data on adenomyosis. Data were extracted from GEO databases (GSE190580 and GSE157718). The GSE190580 dataset shows RNA sequencing of eutopic endometrium and myometrium from women with and without diffuse adenomyosis in the proliferative phase of the menstrual cycle. The GSE157718 dataset refers to transcriptome analysis of eutopic endometrium stromal cells by RNA-sequencing in patients with adenomyosis.

**Figure 2:**
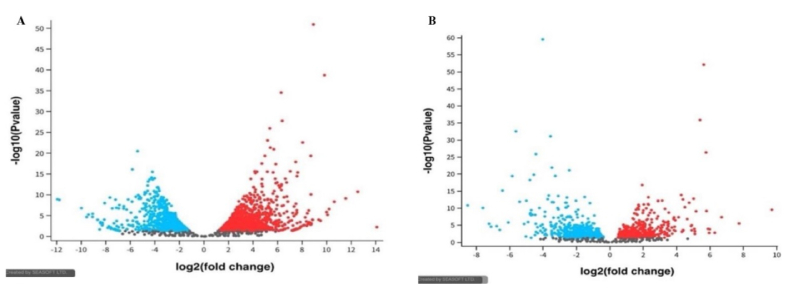
Differential gene expression in adenomyosis: A) Genes from the GSE190580 dataset. B) Genes from the GSE157718 dataset.

Figure 2 display the volcano plots of DEGs identified from GSE190580 and GSE157718 datasets, respectively, illustrating significant upregulation and downregulation of inflammation-related genes in adenomyosis compared to controls. The blue dots represent genes significantly affected by normal control. These genes likely play roles in maintaining normal endometrial physiology, including cellular homeostasis, immune regulation, and tissue repair. The red dots represent genes affected considerably by adenomyosis. Genes upregulation in GSE190580 (blue color), genes upregulation in GSE157718 (red color), and genes with the non-significant changes in expression (grey color). Positive log2 (fold change) values indicate upregulation in gene expression, while negative values indicate downregulation. The y-axis represents the -log10 (Padj) value, which shows the statistical significance of the observed changes in gene expression.

**Figure 3:**
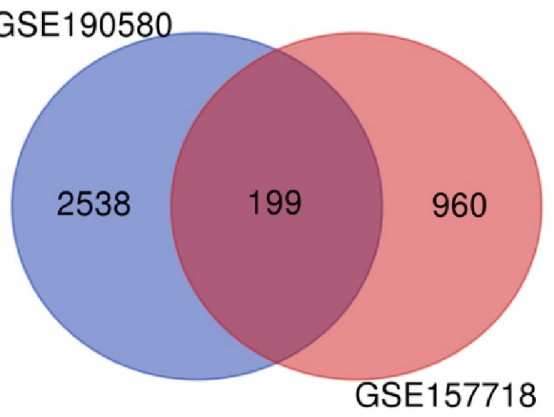
Venn diagram of GSE190580 and GSE157718 genes.

Figure 3 displays a total of 199 overlapping genes. Utilizing ShinyGO for analysis unveiled the functions of these shared genes, of which 199 are linked to adenomyosis. From the GSE 190580 dataset, 2538 unique genes were identified, while from GSE 157718, 960 distinct genes were obtained, without any overlap between the 2 datasets.

**Figure 4:**
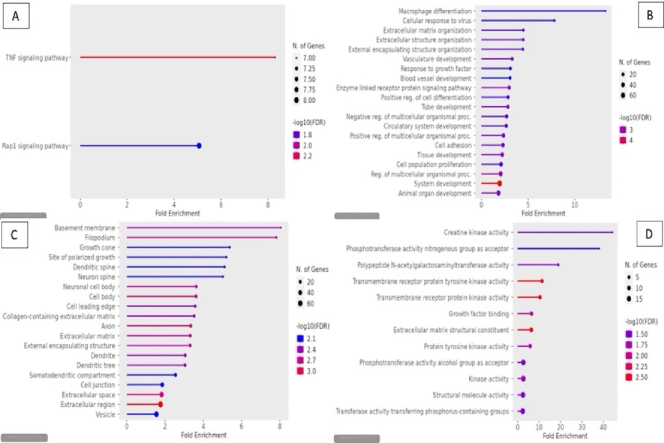
Enriched pathways and functional gene annotations in adenomyosis. A) KEGG. B) GO biological process and ECM organization. C) GO cellular component. D) GO molecular function. The dot plots use a color gradient to represent statistical significance, where red indicates the most significant enrichment (based on -log10 [FDR]). The size of each dot corresponds to the number of genes involved in each pathway.

Pathways involved in adenomyosis are presented in figure 4. Our findings demonstrated that the TNF signaling pathway is the one that most affects adenomyosis. Based on the p-adj FDR value, the adenomyosis pathway image -Log10 (FDR) scale indicates the degree of statistical significance of the pathway analysis. The TNF signaling pathway had the highest -Log10 (FDR) value, which is 2.2 points.

The gene ontology (GO) biological process enrichment analysis depicted in the figure highlights multiple biological processes that may be associated with adenomyosis. Notable processes include macrophage differentiation, extracellular matrix (ECM) organization, cellular response to virus, and vasculature development. Macrophage differentiation shows the highest enrichment, suggesting a strong role of immune cells in the inflammatory responses in adenomyosis. Macrophages are key players in tissue remodelling, inflammation, and fibrosis, all of which are critical in the abnormal uterine growth seen in adenomyosis (15).

The GO cellular component analysis identified key cellular structures associated with DEGs, highlighting several notable findings. Basement membrane and filopodium are among the most enriched components, with fold enrichment values close to 8, suggesting a high concentration of DEGs involved in these structures.

The GO molecular function enrichment analysis, visualized in figure 4, highlights several significant molecular activities associated with endometrial tissues from adenomyosis patients. Notably, “creatine kinase activity" and “phosphotransferase activity, nitrogenous group as acceptor" exhibit the highest fold enrichment, suggesting a strong involvement in energy metabolism and phosphorylation.

## 4. Discussion

This study provides a comprehensive analysis of the transcriptomic landscape of adenomyosis, focusing on inflammation-related pathways and key biomarkers. Our analysis revealed significant dysregulation in the TNF signaling pathway and Rap1 signaling pathway, both of which were highly enriched in adenomyosis samples compared to controls. These findings suggest that persistent inflammation plays a crucial role in the pathogenesis of adenomyosis.

### KEGG

Transcriptomic examination of endometrial tissues from individuals with adenomyosis has revealed important revelations regarding the molecular processes that drive inflammation in this disorder, potentially pinpointing important markers linked to inflammatory responses. The depicted bioinformatic assessment in the illustration emphasizes 2 primary signalling pathways -the TNF signalling pathway and the Rap1 signalling pathway. The enrichment of both pathways implies their participation in the development of adenomyosis. The TNF signalling pathway demonstrates more significant enrichment and encompasses a larger number of genes, underscoring its notable contribution to the inflammatory cascades linked to adenomyosis. TNF-α is a widely recognized cytokine with pro-inflammatory properties that play a crucial role in regulating inflammation and the immune system. In the context of adenomyosis, persistent inflammation has been consistently linked to the abnormal infiltration of endometrial cells into the myometrium, resulting in tissue damage and disease advancement. Research indicates that TNF-α signalling can stimulate inflammatory processes, leading to the misplaced presence of endometrial tissue, a characteristic feature of adenomyosis. Various studies have underscored the role of TNF-associated genes and molecules in inflammatory disorders, including adenomyosis. For instance, proteins like TNF-alpha-induced protein 6 (TNFAIP6) and TNF-alpha-induced protein 6 (TNFAIP3) have been identified as players in modulating inflammatory reactions and immune equilibrium. The disruption of TNF signalling components in adenomyosis could contribute to the inflammatory environment observed in affected endometrial tissues (16).

The Rap1 signalling pathway, while exhibiting lower fold enrichment, still has notable involvement. Rap1 is a small GTPase that regulates various cellular functions, including cell adhesion, proliferation, and migration. In adenomyosis, aberrant cell invasion and tissue remodelling are crucial processes contributing to disease manifestation, and Rap1 may play a key regulatory role in these mechanisms (17).

### GO biological process 

The results of this GO biological process analysis provide insights into the molecular pathways involved in adenomyosis, offering new avenues for biomarker discovery. The enrichment of macrophage differentiation, ECM organization, and vasculature development closely correspond with the established pathophysiology of adenomyosis, which prominently features inflammation, fibrosis, and irregular angiogenesis (18). Macrophages play roles in immune surveillance and tissue repair, and their overactivation in adenomyosis can contribute to the chronic inflammatory state. Targeting macrophage-related biomarkers, such as specific cytokines (e.g., TNF-α, IL-6) or macrophage surface markers (e.g., CD68), could provide diagnostic tools or therapeutic targets for adenomyosis (19). As ECM remodelling is critical in tissue invasion and fibrosis, investigating ECM-related molecules such as collagen, fibronectin, and matrix metalloproteinases (MMPs) could uncover potential biomarkers for adenomyosis. Increased MMP levels have been associated with the invasive tendencies of adenomyotic cells, making them prime candidates for future research (20).

### GO cellular component

The results from the GO cellular component analysis underscore the complexity of adenomyosis at the cellular level. The significant enrichment of genes associated with the basement membrane, filopodia, and ECM suggests that cellular adhesion and tissue remodelling play crucial roles in the pathogenesis of adenomyosis. These mechanisms are recognized for their role in the invasive behaviour of endometrial tissue into the myometrium, a characteristic feature of adenomyosis. The identification of various components associated with neurons, such as the axon and neuronal cell body, supports the growing body of evidence that nerve involvement is critical in adenomyosis, particularly in mediating the chronic pain experienced by patients. This aligns with recent studies showing increased nerve fibre density in adenomyotic lesions. The enriched presence of dendritic spines and growth may also imply alterations in synaptic plasticity, which might further contribute to aberrant nerve signalling and pain perception. Moreover, the enrichment in ECM-related components, such as collagen-containing ECM and external encapsulating structure, highlights the potential role of fibrosis and tissue rigidity in adenomyosis. This observation is consistent with other fibrotic disorders and could serve as a target for novel therapeutic interventions. The identification of enriched ECM elements in this investigation corresponds with existing data indicating the dysregulation of MMPs in adenomyosis, leading to abnormal tissue breakdown and accumulation (20).

### GO molecular function

The GO molecular function enrichment analysis highlights crucial molecular activities associated with adenomyosis, including creatine kinase activity, phosphotransferase activity, and protein tyrosine kinase activity. These results indicate that energy metabolism and signal transduction play significant roles in the condition. Dysregulation of creatine kinase likely affects cellular energy balance, which may exacerbate inflammation through metabolic stress.

The enrichment of protein tyrosine kinase and growth factor binding implies dynamic signalling in ECM remodelling, a hallmark of adenomyosis. Furthermore, the increased activity of polypeptide N-acetylgalactosaminyltransferase and other phosphotransferases indicates that glycosylation processes, which are critical in immune responses, are altered in adenomyosis. Glycosylation impacts protein configuration and signalling, especially in inflammatory pathways. These altered metabolic and signalling processes support the hypothesis that inflammation is a central feature of adenomyosis pathogenesis (21).

### Genes implicated in the inflammatory processes of adenomyosis

Our transcriptomic analysis identified TNFAIP6, matrix metalloproteinase 7 (MMP7), TNFAIP3, leukemia inhibitory factor (LIF), and serum and glucocorticoid-regulated kinase 1 (SGK1) as the top 5 genes implicated in the inflammatory mechanisms of adenomyosis. These genes are strongly associated with the dysregulation of immune responses, ECM remodelling, and tissue inflammation, all critical features of the disease. TNFAIP6 plays a crucial function in maintaining the stability of the ECM and controlling inflammatory reactions, potentially influencing the fibrotic changes seen in adenomyotic lesions. It is induced by TNF-alpha, a key pro-inflammatory cytokine, which has been heavily implicated in adenomyosis pathology. Additionally, TNFAIP6 plays a role in immune modulation and tissue repair, processes that are dysregulated in adenomyosis (22). MMP7 is a MMPs responsible for ECM degradation, a crucial step in the invasion of endometrial cells into the myometrium. Its overexpression is associated with increased tissue remodelling and may promote the invasive characteristics of adenomyosis (23).

TNFAIP3, a negative regulator of NF-κB signalling, regulates the inflammatory reaction and is essential for managing excessive inflammation. Dysregulation of TNFAIP3 may lead to the prolonged inflammatory state seen in adenomyosis (24). LIF is primarily involved in regulating the immune environment in the endometrium, particularly in processes like implantation and inflammation (25). SGK1 is involved in cellular stress reactions and is linked to inflammatory pathways. SGK1 also modulates cell survival, apoptosis, and tissue repair, making it a significant factor in the disease's chronic nature (26).

The clinical relevance of the identified biomarkers lies in their potential to revolutionize the diagnosis and management of adenomyosis. Biomarkers such as TNFAIP6, MMP7, TNFAIP3, LIF, and SGK1, which were identified in this study, could serve as non-invasive diagnostic indicators, enabling earlier detection compared to traditional imaging or histopathological methods. For example, MMP7 has been associated with ECM degradation, a hallmark of endometrial invasion, and could potentially be measured in patient serum as an indicator of disease severity. TNFAIP3, a negative regulator of inflammation, might predict the inflammatory status and treatment response, especially in patients undergoing hormonal therapy. Additionally, LIF and SGK1, known for their roles in implantation and immune modulation, may offer insights into fertility outcomes in adenomyosis patients. These markers, if validated in clinical cohorts, could support personalized treatment strategies, monitor disease progression, and minimize the need for invasive diagnostic procedures such as biopsy or hysterectomy.

While this study provides meaningful insights into the transcriptomic landscape of adenomyosis, we acknowledge certain limitations that may influence the interpretation of the results. First, potential confounding factors such as patient heterogeneity (e.g., age, menstrual phase, hormonal treatment history), variations in tissue sampling, or underlying comorbidities were not fully accounted for due to reliance on publicly available datasets, which often lack detailed clinical metadata. These factors may impact gene expression patterns and pathway enrichment outcomes. Second, although ShinyGO 0.77 served as an effective and accessible tool for GO and pathway enrichment analysis in this study, it had limitations. As with other enrichment tools, ShinyGO may not capture all biologically relevant pathways, particularly those with subtle or weak gene expression signals that fall below standard statistical thresholds. Consequently, some disease-relevant mechanisms may remain undetected. However, it is important to acknowledge that bioinformatic predictions, while powerful, are inherently limited by database annotations, pathway curation, and the static nature of in silico analyses. Not all statistically enriched pathways necessarily translate into functional or causal mechanisms in vivo. Therefore, while our findings provide a valuable framework for understanding the molecular landscape of adenomyosis, further experimental validation at the protein and cellular level is essential to confirm the functional relevance of these biomarkers. The study's limited sample size and focus on specific tissues could also constrain the applicability of results, underscoring the need for additional investigations involving larger participant groups and diverse tissue samples to ensure thorough validation.

Future studies should consider incorporating additional data preprocessing steps and advanced computational approaches to improve the resolution of transcriptomic analyses in adenomyosis. Techniques such as batch effect correction, refined normalization (e.g., variance stabilizing transformation), and dimensionality reduction methods like principal component analysis or uniform manifold approximation and projection may help reveal subtle gene expression patterns and reduce technical noise. These strategies could enhance biomarker discovery and deepen our understanding of the molecular mechanisms underlying adenomyosis.

## 5. Conclusion

Transcriptomic analysis of endometrial tissues from individuals with adenomyosis has provided crucial insights into the underlying molecular processes driving inflammation in this disorder, potentially revealing significant markers associated with inflammatory responses. The bioinformatic assessment highlighted the TNF and Rap1 signalling pathways as enriched in adenomyosis, emphasizing their roles in disease development. TNF-alpha is notably linked to the abnormal invasion of endometrial cells into the myometrium. The GO biological process and cellular component analyses shed light on key pathways and cellular mechanisms involved in adenomyosis pathogenesis, including macrophage differentiation, ECM organization, and nerve-related components. Identified genes like *TNFAIP6, MMP7*, *TNFAIP3*, *LIF*, and *SGK1* underscore their roles in immune responses and tissue inflammation. While limitations exist, such as sample size constraints and reliance on transcriptomic data, further research with larger cohorts and diverse samples, along with experimental validation, is essential for confirming these findings, advancing our understanding of adenomyosis mechanisms, and increasing their therapeutic significance.

##  Data Availability

Data supporting the findings of this study are available upon reasonable request from the corresponding author.

##  Author Contributions

D. Darmawi, M. Yusuf, and F. Febriani designed and conducted the trial. M. Sianturi and A. Syaifulanwar provided scientific and technical support. D. Darmawi, L. Muhammad Irham, and W. Adikusuma supervised the study. M. Sianturi and A. Syaifulanwar carried out bioinformatic analyses, interpreted the findings and drafted the manuscript. D. Darmawi, M. Yusuf, F. Febriani, L. Muhammad Irham, R. Tiara Fani, and W. Adikusuma critically revised the manuscript. All authors read and approved the final version of the manuscript.

##  Conflict of Interest

The authors declare that there is no conflict of interest.
